# Structure Fabrication on Silicon at Atomic and Close-To-Atomic Scale Using Atomic Force Microscopy: Implications for Nanopatterning and Nanodevice Fabrication

**DOI:** 10.3390/mi13040524

**Published:** 2022-03-26

**Authors:** Paven Thomas Mathew, Wei Han, Brian J. Rodriguez, Fengzhou Fang

**Affiliations:** 1 Centre of Micro/Nano Manufacturing Technology (MNMT-Dublin), University College Dublin, D04 V1W8 Dublin, Ireland; paven.mathew@ucdconnect.ie (P.T.M.); wei.han@ucd.ie (W.H.); 2 Shanghai Engineering Research Centre of Ultra-Precision Optical Manufacturing, Fudan University, Shanghai 200433, China; 3 School of Physics, University College Dublin, D04 V1W8 Dublin, Ireland; brian.rodriguez@ucd.ie; 4 Conway Institute of Biomolecular and Biomedical Research, University College Dublin, D04 V1W8 Dublin, Ireland; 5 State Key Laboratory of Precision Measuring Technology and Instruments, Laboratory of Micro/Nano Manufacturing Technology (MNMT), Tianjin University, Tianjin 300072, China

**Keywords:** atomic force microscopy, atomic and close-to-atomic scale manufacturing, silicon, manufacturing III, semiconductors

## Abstract

In this paper, the atomic-scale structure fabrication on Si (100) substrate using atomic force microscopy (AFM) with the aid of electrochemical and mechanical processes in a humid environment and under ambient conditions is studied. The local oxidation patterns are formed using platinum-coated tips with the aid of bias applied to the tip-substrate junction, and direct removal has been achieved using single crystal diamond tips, enabling the structure fabrication at the atomic and close-to-atomic scale. The depth and height of the etched trenches reached about 1 nm, which provides an approach for the fabrication of atomic-scale electrodes for molecular device development. Furthermore, material removal close to about three silicon atoms (~3.2 Å) has been achieved. This is important in molecular device fabrication. A detailed comparison among the nanopatterns and the material removal over bare and hydrofluoric acid (HF) treated silicon substrates is provided. This comparison is useful for the application of fabricating atomic-scale electrodes needed for the molecular electronic components. A deep understanding of atomic-scale material removal can be pushed to fabricate a single atomic protrusion by removing the neighbouring atoms so that the molecule can be attached to a single atom, thereby the AFM tip and Si substrate could act as the electrodes and the molecule between them as the channel, providing basic transistor actions in a molecular transistor design. In this paper, platinum-coated and single-crystal diamond tips are used to explain the oxide formations and direct material removal, respectively.

## 1. Introduction

Research on the fabrication of nanochannels using local oxidation and subsequent etching of silicon surfaces have been widely performed [1,2,3,4,5,6,7,8]. Even though this approach has been successfully demonstrated, it can hinder the material removal mechanism by forming a deposit over the etched area. Avouris et al. [3] presented that the oxide can be removed by treating it with an aqueous solution of hydrofluoric acid (HF). However, if the oxide layer is removed using the tip itself, direct etching can be made possible without any additional methods. A direct etching method over silicon is made possible without oxide formation by Yamamoto et al. [9], where, they have used a catalytically active platinum-coated atomic force microscope (AFM) probe for material removal. Apart from that, methods such as ion-beam sculpting with the application of low- and high-energy ion beams find their relevance in structure fabrication on the atomic scale [10,11].

However, understanding the material removal mechanism is of foremost interest. The conventional mechanisms are well established for micro- and nano-scale manufacturing. On the atomic scale, the material removal could be the result of the combination of different mechanisms such as shearing and extrusion. Material deformations such as ploughing and rubbing could be dominant as well. Usually, during machining, the tip or the machine tool contacts the substrate, and the chips are formed by mechanical shearing [12,13,14]. In the shearing process, due to the compression of the substrate atoms by the tool edge atoms, the substrate atoms are pushed upwards, forming the chips [15]. On the other hand, in extrusion, a plastic deformation is formed beneath the machined surface, and only a few chips will be formed through the extrusion mechanism. The ploughing mechanism is not desirable for machining purposes because it can cause scratches on the machine’s surface, making it less efficient [15]. Even though extrusion is most favored with less chip formation, the actual machining processes at the atomic scale are still not discovered.

Molecular dynamic simulations provide a visual understanding of the principles happening at the atomic scale [16,17,18,19]. Recent studies have shown that the material removal at the atomic scale is mainly caused by the displacement of the substrate atoms [20]. The rake angle, tool edge radius, and the atomistic sizing effects also play major roles [21,22,23,24]. In order to consistently remove atoms from the topmost layer of material, an atomically precise machining tool should be approached with optimum parameters such as tip force (F_T_), tip bias, tip velocity (V_T_), and tip mechanical properties. AFM tips are widely used as the machining tool to understand the fundamentals of structure fabrications at the atomic scale.

As silicon is well known for its applications in the semiconductor industry, more precise and consistent material removal should be conducted over it. Apart from that, silicon in its (100) orientation is best suited for the structure fabrication studies as the Si/SiO_2_ state density is lower than the other orientation, yielding higher carrier mobility when used for nanodevice fabrications. The research gap here is the minimum depth or the heights of atomic patterns that can be achieved to realize structure fabrication using AFM-based electrochemical and mechanical scratching methods using an AFM tip. A removal depth of 1.4 Å is achieved through mechanochemical methods [25], but the electrochemical and mechanical mechanisms are unknown, which forms the motivation for this study—with the mechanisms elucidated, it becomes possible to deterministically engineer removal depth. Moreover, the comparison between material removal before and after the oxide layer removal can give a better understanding of the removal mechanism. The influence of the oxide layer is beneficial for fabricating different structures and patterns over the silicon surface. These structures can be used as references for validating the integrated circuit (IC) chip electronic component actions such as the electronic transmission and the conductivity to use these materials as transistors or other devices in their miniaturized versions. Apart from Si, materials such as, highly oriented pyrolytic graphite (HOPG), gold, silicon carbide and van der Waals materials such as transition metal dichalcogenides (TMD) are also potential candidates for atomic-scale manufacturing studies using AFM. As a step towards this, in our recently published article, we have shown that a single atomic layer removal over HOPG can be achieved [26].

In this paper, a detailed study on the oxide formation on a silicon surface, with platinum coated tip, while scratching when given a tip bias and the material removal using a diamond tip, using AFM are identified. The differences while using platinum coated and diamond tips over silicon surface is provided, giving information on the structure fabrications in the atomic scale.

## 2. Materials and Methods

**Figure 1:**
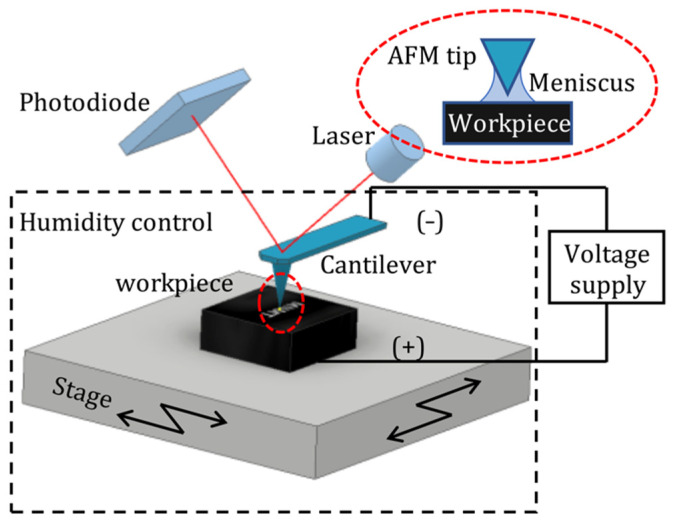
Schematic diagram of AFM-based electrochemical etching apparatus with a relative humidity control environment.

The electrochemical etching mechanism is performed using a commercially available AFM system (MFP-3D, Olympus, Asylum Research, Santa Barbara, CA, USA). A silicon-based AFM probe coated with conductive PtIr_5_ (PPP-EFM, Nanosensors, Neuchatel, Switzerland) and single-crystal diamond probes (Adama probes, AD-40-AS, Bruker France, Wissembourg Cedex, France) are used, both in contact mode for the machining and in amplitude modulation for imaging. The spring constant of the PPP-EFM tip was found to be 2.9 N/m and that of the diamond tip to be 20.7 N/m, with an uncertainty of ±10% [27,28], with a nominal tip radius of 20 nm and 10 nm, respectively. Silicon in its native oxidized and HF-treated states (48% HF acid, ThermoFisher scientific, Loughborough, UK) were used for the experiments. Bare silicon was stored in ambient conditions and cleaned by sonicating in acetone and isopropanol for 30 min at 25 °C temperature and then rinsing it in deionized (DI) water. The second substrate was further cleaned by removing the native oxide by dipping it in 10% aqueous HF solution for 10 s. The native oxide layer on the surface is considered to be less than 2 nm in height [29]. The oxide layer will grow to a thickness of ~2–3 Å during water rinse after the HF dip. Experiments on HF-treated silicon substrates are performed within 1 h of the dip, so that it can be confirmed that the lithography is performed on the silicon surface, rather than on the oxide layer. Figure 1 shows the schematic diagram of AFM-based electrochemical etching apparatus with a relative humidity control environment.

**Figure A1:**
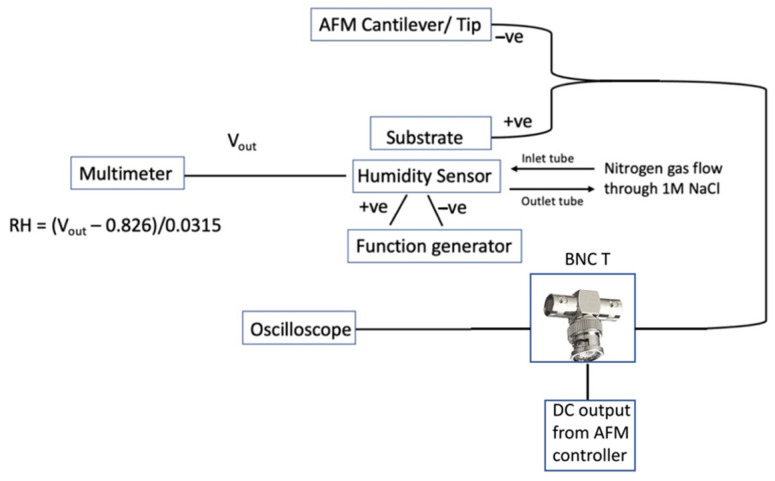
The schematic representation of the external circuitry provided to apply bias to the AFM tip and substrate. BNC: Bayonet Neill − Concelman coaxial cable connector; RH: Relative Humidity.

The humidity conditions were provided by bubbling dry nitrogen gas over 1M NaCl solution (ThermoFisher scientific, Loughborough, UK), which accounted for relative humidity (RH) ranging from 75% to 90%. The humidity range was monitored with the aid of a humidity sensor (HIH—4000 series), a function generator with its output set at 6V DC and a multimeter. A separate circuit was provided to connect the tip to the negative terminal and the substrate to the positive terminal. The voltage applied is monitored using an oscilloscope (Hitachi V-1560, Tokyo, Japan). The schematic of the external circuitry is given in Figure A1. The experiments were also conducted in ambient air conditions with a room humidity of 22–38%. The temperature-controlled lab in which the experiments were performed was monitored to have a temperature of 20 ± 1 °C.

## 3. Results and Discussion

### 3.1. Scanning Probe Lithography (SPL) over Bare Silicon (with Native Oxide)

#### 3.1.1. With Platinum Coated Tips

The formation of oxidation over the silicon substrate in the presence of oxygen is well-known. The tip forms a water meniscus in contact with the adsorbed water layer at the silicon surface. The oxide deposition can be controlled with many parameters such as tip velocity, applied voltage, humidity, and applied tip force. The water reacts with silicon to form hydroxides and oxides, as given in Equations (1) and (2).

(1) $$2H_{2}O+2e^{-}\;\to \;H_{2}+2OH^{-}$$

(2) $$Si+2OH^{-}\;\to \;SiO_{2}+2H^{+}+4e^{-}$$

**Figure 2:**
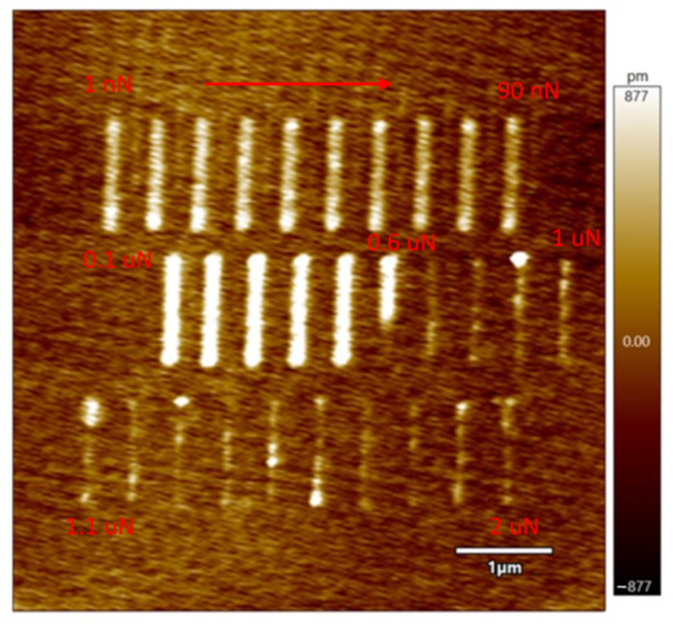
The lines scratched with different tip force: Top lines are the forces ranging from 1 nN to 90 nN with an increment of 10 nN. Middle lines are the forces ranging from 0.1 μN to 1 μN with an increment of 0.1 μN. Bottom lines are the forces ranging from 1.1 μN to 2 μN with an increment of 0.1 μN.

The thickness of the oxide layer normally ranges from 1.5 nm to 2 nm. When lines were scratched over the bare silicon substrate, under normal room conditions with a humidity of 36%, local anodic oxidation (LAO) occurred. The force with which the lines were drawn is 0.1 μN. This force is the minimum force used for fabricating nanostructures over silicon substrate using Au-coated and platinum-coated AFM tips [30]. To find an optimized force value with the current set-up, using 7 V tip bias and 1 μm/s tip velocity, different lines are drawn with different force values ranging from 1 nN to 2 μN, as shown in Figure 2. Here, the top row represents the lines drawn with forces ranging from 1 nN to 90 nN. The middle row represents force values from 0.1 μN to 1 μN, and the bottom row represents the force values from 1.1 μN to 2 μN.

**Figure 3:**
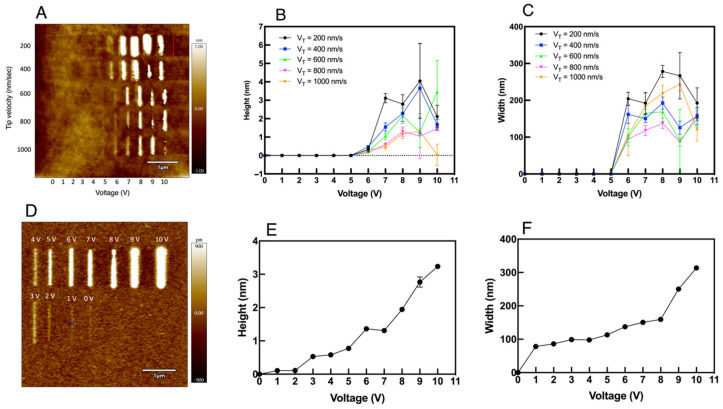
The lines drawn on the surface under different humidity conditions: (**A**) The formation of oxide deposits with different tip velocity and voltage (**B**) The height vs. tip bias and (**C**) Width vs. tip bias of the lines scratched on the silicon surface. F_T_ = 0.1 μN. RH is 36% (**D**) The lithographical lines drawn on the substrate with different voltage with a relative humidity of 75% and V_T_ = 1 μm/sec. F_T_ = 0.1 μN (**E**) The height vs. tip bias and (**F**) width vs. tip bias characteristics.

From Figure 2, it is clear that the lines are very well distinct and controlled for the force values starting from 0.1 μN. Apart from that, the LAO happened for the lines with force values as low as 1 nN, and it started to be limited from 0.6 μN. The lines are faded from 0.6 μN to 2 μN. Hence, the upper threshold force value for the LAO to stop is 0.6–0.7 μN. With this optimal force, different lines are drawn with different tip voltages and tip velocities. Figure 3 shows a comparison between the effects of oxide formation with low RH (See Figure 3A–C) and high RH (See Figure 3D–F). From the figures, the height and width of all the scratched lines are measured by considering five points within each line, spreading from top to bottom, and the standard deviation is measured to provide the uncertainties in the values. In Figure 3A, it can be seen that the oxidation thickness is higher and wider when the speed is low. At 200 nm/s, the height of the deposit has gone just above 4 nm, and with higher speed, the height is just above 1 nm. This is due to the difference in points of contact between the tip and substrate atoms with varying speeds. With lower speed, more points of contact can be achieved, facilitating the formation of thicker and wider oxides, whereas, with higher speeds, few atoms take part in the oxide formation. A sudden increase in the deposits can be seen from 6 V, and it becomes very much distinct from 7 V. This is because of the increased electric field strength, as shown in the earlier works of Cabreta and Mott [31], where the field lowers the activation energy for the ionic species to transfer through the tip-substrate junction. Even though an inconsistent increment and decrement in height and depth can be seen for different tip velocities, V_T_ at 1 μm/s can be seen causing LAO with a consistent increase in height and width till 9 V, after that, a drop in both variables could be observed at 10 V, as shown in Figure 3B,C. Furthermore, LAO starts to be clearly visible from 7 V for all the tip velocities. Hence, the formation of oxide deposits has a great influence on V_T_ and the voltage applied, but a threshold voltage of 6–7 V is required for the deposits to be seen on the substrate, with a clear and distinct formation at 7 V. This is due to the electric field strength not sufficient enough for the reaction to take place below 6 V.

Apart from that, a notable change in the height and width of oxidation, with respect to the decrease in tip velocity, happens after 9 V. This is because of the self-limiting reaction taking place with higher voltage. When the electric field is high, up to 9 V, the oxides are formed with increasing thickness, and this oxide thickness reduces the electric field strength needed for further oxidation and hence reduces the growth rate [1]. With 10 V, the width is almost comparable with different tip velocities. Hence the critical voltage is not strongly affected by the tip velocity after 9 V. This observation could be utilized while performing surface fabrication over silicon.

However, when the experiments were performed in the presence of humidity, the LAO behaved differently. The formation of oxides on the surface became consistent and more pronounced than in the ambient air. Figure 3D shows the different lines drawn on the silicon surface with a humidity range of 75–90%. From Figure 3E,F, it can be seen that there is a consistent increase in the width and height of the deposits occurring on the surface. The threshold to obtain the deposits were 6–7 V under ambient conditions, whereas the deposits start to be clearly visible from as low as 2 V. This indicates that the LAO is largely influenced by the presence of relative humidity. This allows the easy formation of oxides and enhances the formation of a water meniscus at the tip-substrate junction.

Since a well-defined, consistent and controlled pattern occurred at 7 V potential, 1 μm/s V_T_ and 0.1 μN F_T_, all the experiments are performed by applying a 7 V bias to the tip-substrate junction, the substrate being positive and tip grounded, until and unless mentioned otherwise, a V_T_ of 1 μm/s and an F_T_ of 0.1 μN. Hence, 0.1 μN, 7 V tip bias and 1 μm/s V_T_ would be best suited for the structure fabrication over the silicon substrate.

**Figure 4:**
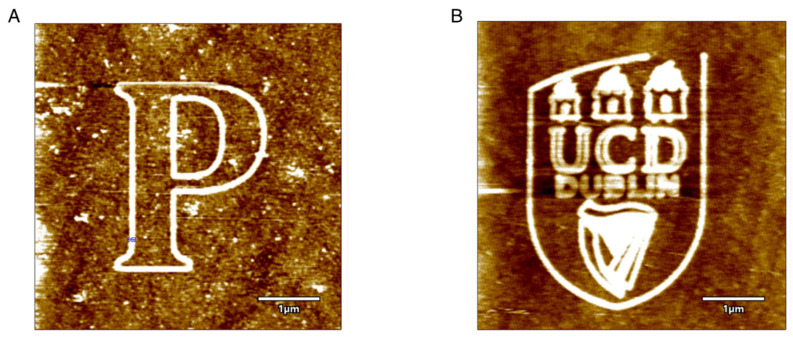
The intrinsic patterns fabricated over silicon substrate: (**A**) Letter ‘P’, and (**B**) UCD logo. F_T_ = 0.1 μN and tip bias of 7 V, RH = 75%, V_T_ = 1 μm/s.

Some of the letters drawn on the surface under the humid environment, with an RA of 75%, is shown in Figure 4. The letters can be seen well defined and clear with comparable thickness throughout. Hence, to conclude, with platinum-coated conductive tips, LAO occurs, and it is influenced by the tip velocity, tip bias, tip force and humidity conditions. With this, structure fabrication with raised patterns can be achieved, whereas material removal at the atomic scale is not possible directly unless, these substrates are treated with HF acid, which is a time-consuming procedure. Furthermore, as mentioned before, with the catalytically active platinum-coated AFM tip, oxides are not formed, and direct material removal is possible. Here, the oxides are formed because of the absence of any kind of catalyst.

#### 3.1.2. With Single Crystal Diamond Tip

**Figure 5:**
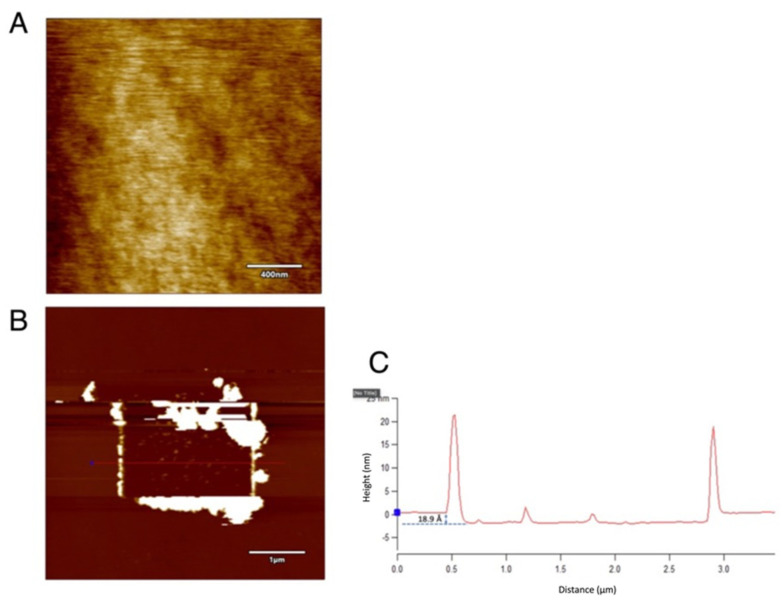
The contact mode scanning over silicon surface using single crystal diamond tip: (**A**) The polished 2 × 2 μm area by contact mode scanning with a force of 2 μN. (**B**) The accumulation of debris on the sides and the (**C**) the line profile showing a depression of 1.89 nm. Tip bias: 0 V and RH ~75%.

In the above section, oxide deposits were formed over the silicon substrate rather than material removal when platinum-coated tips and applied bias were used. When the tip is replaced with a single crystal diamond, under humidity conditions (~75% and room humidity ~25%) and the same parameters mentioned in the previous section, the results are in favor of material removal. If LAO happened with platinum tips, direct material removal was possible with the diamond tip. Figure 5 shows the contact mode scanned image of silicon in 2 × 2 μm area, with a force of 2 μN. This force is employed since it has been used for implementing nanolithography over hard substrates such as silicon using single crystal diamond tips [32]. The region seems clear with no debris. When a tapping mode scanning of 5 × 5 μm area was performed, all dust particles and the oxide deposits can be seen at the boundaries. The line profile shows a depression in the scanned area of approximately 1.89 nm, which ought to be the native oxide layer on the surface that was removed.

From the figure, the debris and the dust particles can be seen accumulated on the sides, but it is more pronounced on the right and bottom sides of the scanned area. This happens because of the geometry of the tip used. The pyramidal shape of the tip, when scanned at 0° angle, the two sides adjacent to the scanning phase, pushes the particles to both sides, and since significantly less debris is formed behind the scanning side, the accumulation is less prominent on the left side. The arrow indicates the direction of the scan. As the native oxide is removed by the contact mode of scanning, the machining can be considered to be performed on the actual silicon surface.

**Figure 6:**
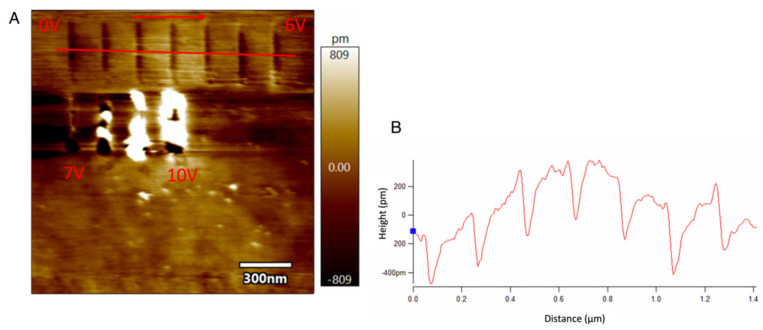
The scratched lines and the line profile with different tip voltage over silicon substrate with diamond tip: (**A**) The scratched lines over silicon using single crystal diamond AFM tip with different tip voltage ranging from 0 V to 10 V with constant velocity (**B**) The line profile of the trenches (non-flattened). F_T_ = 4 μN and the humidity is ~75%.

Figure 6 shows the lines scratched on this cleaned area with a slightly larger F_T_ of 4 μN and different velocities ranging from 0 V to 10 V, with a V_T_ of 1 μm/s. In the figure, stable and consistent material removal is obtained for lower voltages till 6 V. With the voltage higher than 6 V, the surface starts to be damaged. Ploughing takes place, and the plastic deformation causes the surface to bulge around the edges of the scratch. However, the dependence of bias over the diamond tip is almost negligible since the depth and height of these lines are not affected by different voltages. The average depth of the seven trenches covered under the line profile is calculated to be 3.98 Å, which is approximately three silicon atoms thick.

**Figure 7:**
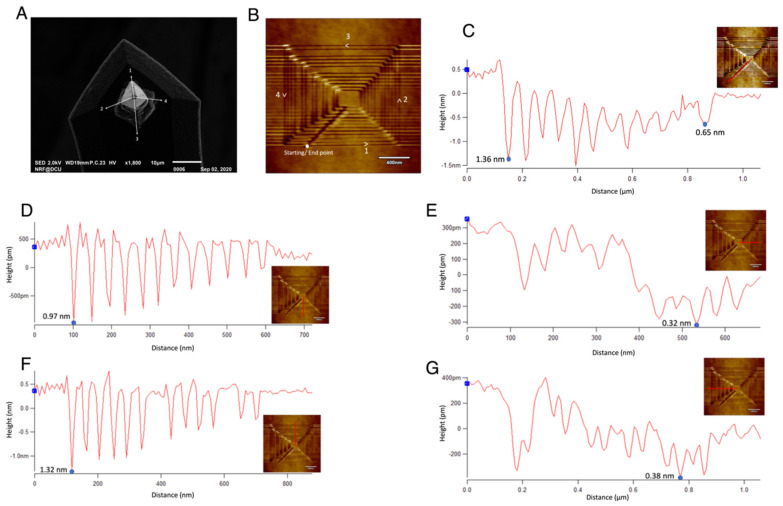
Analysis of different depth rates around the etched concentric square: (**A**) The SEM image of the diamond tip before scratching the silicon surface (**B**) The concentric square etched on the surface indicating the direction of tip motion and the sides of the tip with which the lines were in contact (**C**) The line profile along the starting/end point, (**D**) The line profile along the rightward horizontal section where the AFM tip side 1 was in contact (**E**) The line profile along the upward vertical side where AFM tip side 2 was in contact (**F**) The line profile along the leftward horizontal plane where the AFM tip side 3 was in contact (**G**) The line profile along the downward vertical side of the concentric square where the AFM tip side 4 was in contact. The inset figures on the line profile shows the representation of the lines on each side of the square.

**Figure A2:**
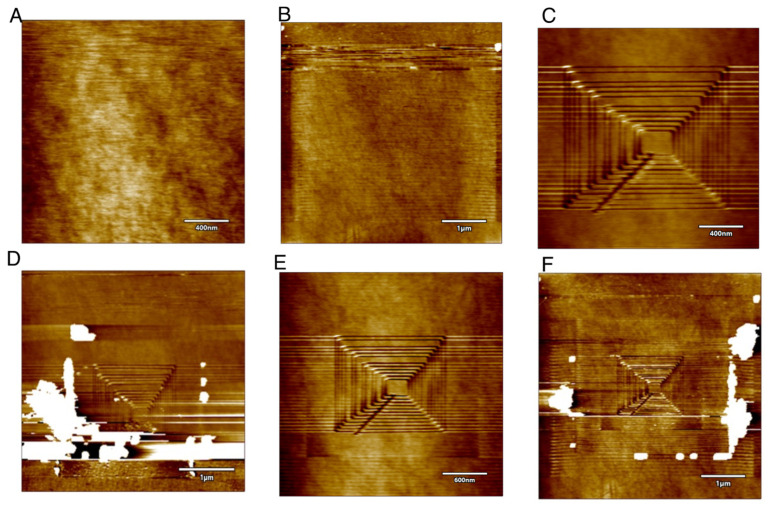
Scratching tests to show the material removal over the silicon substrate. (**A**) Contact mode scanning over 2 × 2 μm area with a F_T_ of 2 μN (**B**) Tapping mode scanning of 5 × 5 μm area (**C**) The concentric circles scratched over the silicon surface with a normal F_T_ of 4 μN (**D**) The accumulation of debris at the boundary of 2 × 2 μm area (**E**) Contact mode scanning over 3 × 3 μm area, where the debris accumulated at the 2 × 2 μm area is swept away (**F**) The displaced debris accumulation at the 3 × 3 μm area boundary, proving the actual materials formed which can be swept away using contact mode scanning, by the AFM tip. Tip bias: 0 V.

To understand more about the influence of material depth with respect to different directions, a concentric square is scratched over the substrate under normal room conditions (room humidity 25–30%), as shown in Figure 7B. A clearer criterion in which the square was scratched is given in Figure A2. From Figure 7B, some of the lines are deeper, and some lines are less prominent. This is because of the tip geometry. The SEM analysis of the diamond tip before scratching is shown in Figure 7A. In the figure, different sides are represented in numbers for an easy explanation of the analysis. The numbers in Figure 7B depicts the same numbers from Figure 7A, through which different sides of the AFM tip passes during the scratching process, shown by the arrows. The scanning is performed at the 0° angle, where the lines are horizontally scanned with side 1 and retraced with side 3 of the tip. As it can be seen, the starting and end point of the square is much deeper, as can be seen from the line profile starting from the centre of the square, shown in Figure 7C. A possible reason for this is the impact of the tip while starting and finishing the scratch at the same point. Another inference is that depth decreases as the size of the square increases. The depth is as deep as 1.36 nm for the smallest and the innermost square, while the outermost square has a depth of merely 0.65 nm. This is because of the time duration of the scratching between the start and end point.

Different sides of the tip seem to vary in removal rates. Side 1 and side 3 causes more materials to be removed from the substrate, whereas side 2 and side 4 causes less removal. This can be because of the horizontal and vertical crystal orientations of the silicon substrate. Material removal can be seen more prominent in the horizontal direction, which is 0° to the scan angle, than the vertical direction, which corresponds to 90° to the scan angle. The materials are removed to a depth less than 1 nm in the case of the vertical direction, reaching as small as 0.32 nm (Figure 7E). The dominant material removal mechanism here is the mechanical processes with chips formed that can be removed or displaced using the contact mode of scanning.

### 3.2. SPL over HF Treated Silicon

#### With Platinum and Single Crystal Diamond Tip

**Figure 8:**
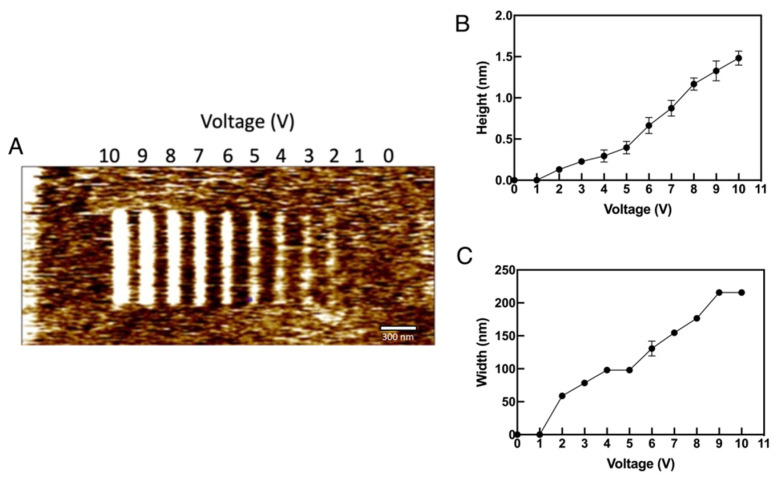
The LAO formation over HF treated silicon: (**A**) The formation of oxide deposits over HF treated silicon with different tip voltage ranging from 0 V to 10 V (**B**) The height vs. tip voltage characteristics and (**C**) The width vs. tip bias graph. F_T_ = 0.1 μN, V_T_ = 1 μm/s and RH ~26%.

In this section, experiments are performed over the silicon substrate dipped in 10% HF solution to remove the native oxide layer over the substrate. When the experiments are performed drawing lines on the surface using a platinum-coated conductive tip, the results are similar to that obtained over the bare silicon, as shown in Figure 8. Distinct oxidation can be seen over the surface for different voltages ranging from 0 V to 10 V, with a V_T_ of 1 μm/s and an F_T_ of 0.1 μN. Hence, it can be concluded that no matter whether there is a native oxide layer or not, oxidation happens with the platinum-coated tip.

The height and width of the oxide deposits are much more consistent and controlled over the HF treated surface, as shown in Figure 8B,C, respectively. From the graphs, it can be seen that with higher potential, the height and width are well pronounced and vice-versa. The experiments on HF treated substrates are performed under normal room conditions because the humidity set-up could facilitate the formation of oxide layers much faster.

**Figure 9:**
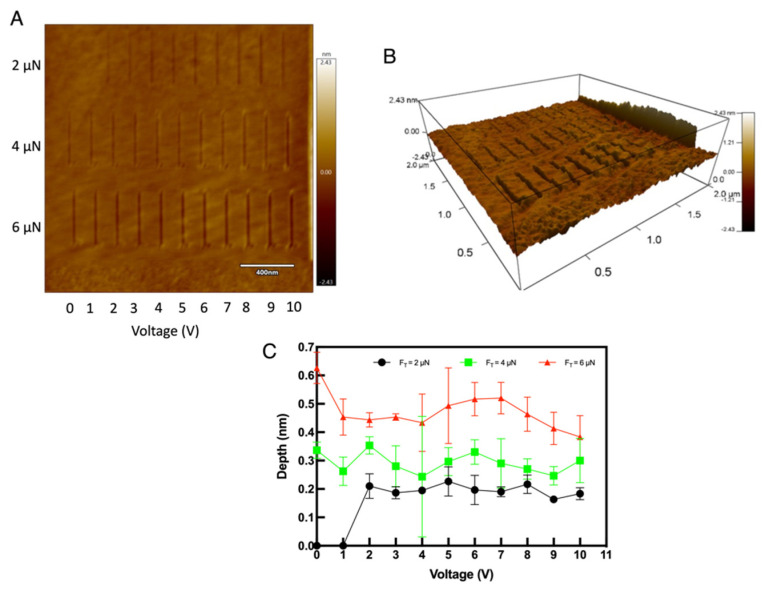
Scratching tests on HF treated silicon: (**A**) The etched lines on the silicon surface with different forces and voltages (**B**) The 3D image of the same where the etched depth can be seen clearly increasing with higher force (**C**) The depth vas voltage graph showing the influence of different tip force.

On the other hand, when the area is subjected to lithography using the diamond tip, material removal is achieved. Figure 9A shows the experiment on HF treated silicon with three force values (2 μN, 4 μN and 6 μN) and the voltages, scratched with 1 μm/s tip velocity and Figure 9B shows the 3D imaging of the same surface. From the figures, it is evident that the material removal is achieved directly over the substrate.

**Figure A3:**
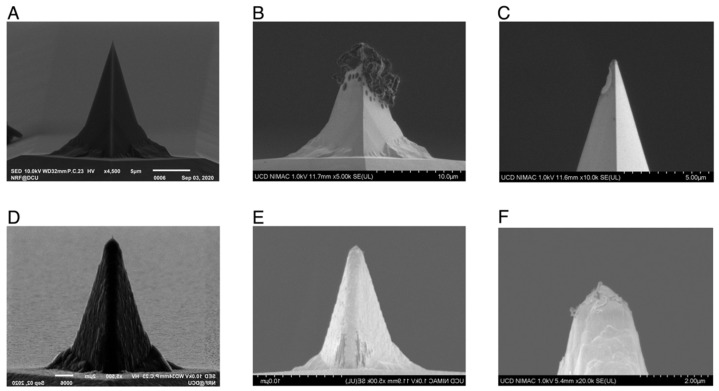
The SEM images of AFM tips. (**A**) Platinum coated tips before experiments (**B**) After experiments with humidity set-up involving the use of NaCl electrolyte (**C**) After experiments in ambient air without any electrolyte (**D**) SEM image of diamond tip before experiment and (**E**) SEM image of diamond tip after experiment in the presence and (**F**) absence of electrolyte. The worn-out tips shown here are imaged after ~12 h of experimentations.

From the graph shown in Figure 9C, the influence of different force values can be elucidated. With lower force, depths as close to a single atomic layer can be achieved. In addition, the depths are almost comparable for all the force values applied here, even though tip bias from 0 V to 10 V has been applied. This again shows that the diamond tips are independent of the bias voltage, which again is favorable for the ACSM procedures. Another observation is that the etched lines are consistent for voltages up to 10 V over HF treated silicon, but for bare silicon, the surface damage could be seen for voltages above 6 V (see Figure 6A). Hence, for a consistent and better-controlled material removal over silicon, HF treated substrates are best suited than the bare silicon. The influence of scratching on both platinum and diamond tips are shown in Figure A3. From the SEM analysis (Appendix C (Figure A3A–F)), diamond tips are best suited over platinum tips for material removal.

## 4. Conclusions

The influence of different parameters on the structure fabrication over silicon (100) substrate using two different AFM tips is studied in detail. It is found that better fabrication and material removal can be achieved on the atomic scale under optimized parameters. With a platinum-coated AFM tip, deposition on the substrate takes place instead of material removal. However, material removal is confirmed with a diamond tip at close to the atomic scale, since a depth of as small as 0.32 nm (3.2 Å) is obtained, which is about three silicon atoms. The formed debris can be swept to the edges by contact mode scanning. The material removal can be controlled by optimizing the parameters, such as tip velocity, tip voltage, and the tip force applied. From the study, a tip bias of 7 V, F_T_ of 0.1 μN and V_T_ of 1 μm/s are most favorable for the fabrication while using the platinum-coated tip. In contrast, tip bias is almost independent while using the single-crystal diamond tips. For diamond tips, an F_T_ of 2 μN and a V_T_ of 1 μm/s, could yield close-to-atomic scale material removal. The relative humidity is highly influential in the structure fabrication, as a smooth and consistent LAO is achieved. The material removal is found to be inconsistent with some depositions/surface damage over bare silicon with a native oxide layer, but it is consistent and smooth over HF treated silicon. Direct material removal is obtained over bare silicon by using the diamond tip, whereas a two-step process is required while using the platinum-coated tips. For atomic-scale manufacturing, diamond tips are confirmed to be best suited over platinum-coated tips. This study forms a foundation for the fabrication of future nanodevices, and its application ranges from the development of miniaturized electronic components to the mass production of atomic-scale IC components leading to the next generation of the manufacturing phase, i.e., Manufacturing III.

## Appendix B. The Scratching of Concentric Square

Figure A2 shows the evidence of direct material removal with the diamond tip under normal room conditions (room humidity 25–30%). Figure A2A is the contact mode scanning over 2 × 2 μm area, and Figure A2B shows the tapping mode scanning over 5 × 5 μm area, which shows the zoomed-out image of the 2 × 2 μm area. No debris was found at the edges of 2 × 2, hence showing that the area is free of dust particles. To obtain large scratched patterns using single scratching, a concentric square was etched in the mentioned 2 × 2 μm area and was scanned in contact mode (Figure A2C). The debris formed as a result of this scratching accumulated along its boundaries, as can be seen from the 5 × 5 μm tapping mode scan shown in Figure A2D. When the above mentioned machined 2 × 2 μm area was further scanned in contact mode over 3 × 3 μm (Figure A2E), the accumulated debris was pushed out to the 3 × 3 μm boundary. This is confirmed by the 5 × 5 μm tapping mode scan (Figure A2F). Hence, this experiment proved that material removal occurred, and the debris formed can be moved with a contact mode scan.

## Appendix C. The Effects of Scratching on AFM Tips

Figure A3 shows the damage caused to the tips after scratching. Figure A3A shows the platinum-coated tip before scratching. It can be seen that the tips were destroyed after the experiments, as shown in Figure A3B. This can be caused due to the removal of platinum coating from the tip surface and could also be the depositions caused due to the NaCl solution used to facilitate the humidity conditions. This conclusion is drawn because no such depositions were seen on the platinum tips when the experiments were performed in ambient air conditions, as shown in Figure A3C. Hence the use of electrolytes affects the geometry of the tips, and prolonged use of the same tip can result in different structures on the substrate. Figure A3D,E show the SEM images of the single-crystal diamond tip before and after the experiments. No observable damage has occurred to the diamond tips, which makes them perfect for durable and longer experimentations. Moreover, no deposits are caused on the tip even in the presence of an electrolyte, as shown in Figure A3F. This could also be convenient for performing investigations without changing the tips too often and in any environment.
